# 1006. Case Study. Staged Coverage for Abdominal Catastrophe

**DOI:** 10.1093/jbcr/irag033.194

**Published:** 2026-04-06

**Authors:** Nicholas Wagner, Alyssa D Brown, Alan Pang

**Affiliations:** Texas Tech University Health Sciences Center, Lubbock, TX; Texas Tech University Health Sciences Center, Lubbock, TX; Texas Tech University Health Sciences Center, Lubbock, TX

## Abstract

**Patient Presentation (age range, injury details, relevant history):**

The patient is a 48 year old male who presented for intractable abdominal pain. He had a large ventral hernia with overlying skin changes. He was taken emergently for exploratory laparotomy, and he was found to have a closed loop bowel obstruction with perforation. Overlying necrotic abdominal wall was excised. The abdominal fascia was unable to be closed appropriately due to the extent of the ventral hernia. The patient was left in discontinuity and an intra-abdominal vacuum-assisted closure (VAC) device was placed. He was taken back to the OR in two days for washout and re-anastomosis with intra-abdominal VAC device placement. On post-operative day 5, we performed an autografting of the abdominal wound with autologous skin suspension and wound VAC placement. After 7 days, his wound VAC was exchanged, and his graft had 90% take.

**Clinical Challenges:**

Abdominal catastrophes are cases that require creative wound closure solutions because of their size and lack of fascia. While an intra-abdominal vacuum-assisted closure device can be helpful, it alone is not a definitive solution for these abdominal wounds.

**Management Approach:**

We present a staged approach consisting of initial abdominal surgery, placement of an intra-abdominal vacuum-assisted closure device, autografting with an autologous skin cell suspension, and follow-up component separation and definitive closure/hernia repair. By staging the coverage of these, we can decrease the time to discharge from ICU and maintain grafting efficacy.

**Outcomes:**

The patient was able to be discharged to inpatient rehabilitation. No adverse events were attributed to the autograft with autologous skin cell suspension over the open abdominal wound.

**Lessons Learned:**

Catastrophic abdominal wounds that are unable to be primarily closed are a clinical conundrum. A staged approach of initial abdominal surgery, placement of an intra-abdominal vacuum-assisted closure device, autografting with an autologous skin cell suspension, and future component separation and definitive closure/hernia repair. By staging the coverage of these, we can decrease the time to definitive closure and ICU stay time.

**Applicability to Practice:**

We propose a staged approach to catastrophic abdominal wounds consisting of initial abdominal surgery, placement of an intra-abdominal vacuum-assisted closure device, autografting with an autologous skin cell suspension, and future component separation and definitive closure/hernia repair.

